# Rippling Associated with Pre-Pectoral Implant Based Breast Reconstruction: A New Grading System

**DOI:** 10.29252/wjps.8.3.311

**Published:** 2019-09

**Authors:** Raghavan Vidya, Fahad Mujtaba Iqbal, Hilton Becker, Olga Zhadan

**Affiliations:** 1 Department of Oncoplastic Breast Surgery, New Cross Hospital, Wolverhampton, UK;; 2 Academic foundation Doctor, St. George’s Hospital, London, UK;; 3 Clinic of Plastic Surgery, Boca Raton, Florida, USA;; 4 Clinical Biomedical Science, Charles E. Schmidt College of Medicine, Florida Atlantic University, Boca Raton, Florida, USA;; 5 Department of Plastic and Reconstructive Surgery, Cleveland Clinic Florida, Weston, Florida, USA

**Keywords:** Breast, Implant, Reconstruction, Complication, Rippling, Lipomodelling

## Abstract

**BACKGROUND:**

The incidence of breast cancer and immediate breast reconstruction is on the rise particularly in the US and Western Europe. Over the last decade, implant based breast reconstructions have gained popularity. The prepectoral breast reconstruction has emerged as a novel technique, minimally invasive, preserves the chest wall anatomy while restoring body image. However, implant rippling appears to be an adverse effect associated with this technique.

**METHODS:**

We have described a new grading system for rippling following prepectoral implant breast reconstruction and discussed its management. We then evaluated the new grading system in our practice.

**RESULTS:**

We looked at the first 50 consecutive patients who underwent prepectoral implant based breast reconstruction. In our experience, 45 patients (90%) had grade 1, 3 patients (6%) had grade 2, 1 patient (2%) had grade 3 and 1 patient (2%) had grade 4 rippling. The observed rippling was seen more often in patients with low BMI<20 and in those who had poor subcutaneous fat preoperatively (pinch test<2 cm).

**CONCLUSION:**

Prepectoral implant based breast reconstruction adds a whole new dimension to breast reconstruction. However rippling can be an undesired adverse effect associated with this technique and patients need to be informed.

## INTRODUCTION

Implant-based breast reconstructions account for 40-60% of all breast reconstructions performed in the UK and approximately 75% in the United States.^1^ Prepectoral breast reconstruction is once again becoming popular with the development of new meshes and implants. The current technique of creating a new breast in the pre-pectoral plane usually involves ex-vivo coverage of the breast implant with a mesh and subsequent attachment to the chest wall, preserving both the pectoralis major and serratus anterior muscles. As the breast remains in its anatomical plane animation deformity is prevented,^2^ postoperative pain is reported to be lower, and shoulder function is not impaired.^3^^,^^4^

Implant rippling, however, remains a large concern.^5^ We aim to provide a new grading system for rippling with prepectoral implant breast reconstruction in order to guide its management. The prepectoral mesh forms an internal bra with the mesh implant wrap, which in turn is secured to the chest wall. Biological meshes integrate through collagen remodelling, which ultimately integrate with host tissue becoming vascularised.^6^ The collagen matrix in biological grafts aids in remodelling and new collagen deposition.^7^ Synthetic meshes create a scaffold and promote fibrous tissue growth to cover for the implant. Integration occurs via a fibroblastic reaction alongside a mild inflammatory response.^8^

Over time, and under the weight of the implant, the upper part of the prepectoral reconstruction atrophies. This, coupled with thinning of the collagen in the skin, can result in visible implant rippling. Indeed, subcutaneous cover influences the degree of rippling and is often less prevalent in those with a high body mass index (BMI).^8^

## MATERIALS AND METHODS

**Fig. 1:**
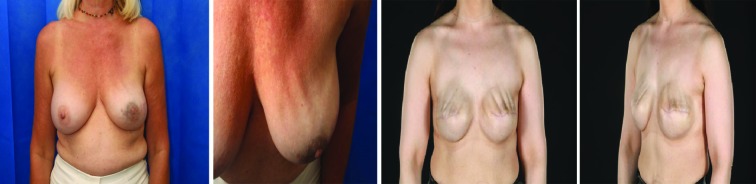
Demonstrating the grading of rippling. Grade 3: Moderate degree of rippling seen at both rest and exercise. Grade 4: Persistent rippling causing gross deformity. Bra can cause impressions on the breast as seen in this patient

We have tabulated the degree of rippling (Table 1) to aid with management. The degree of rippling has been graded 1-4. The photographic illustration to demonstrate the degree of grading are enclosed (Figure 1). We then evaluated the new grading system in our practice. All patients who underwent prepectoral implant based breast reconstruction surgery from Sept 2014 to 2017 were included in the study. The approval was obtained from the institutional review committee.

**Table 1:** A novel grading system for rippling in implant-based breast reconstruction

**Grade**	**Definition**	**Management**
1	No evidence of rippling seen both at rest and with movement	No intervention needed
2	Mild rippling is felt but not visualised both at rest and with movement	Offer intervention
3	Moderate rippling visualised with movement and at rest	Needs intervention
4	Severe - persistent rippling causing gross deformity both at rest and with movement	Needs intervention

Patients were selected for this new procedure according to the Association of Breast Surgery and the British Association of Plastic, Reconstructive and Aesthetic Surgeons’ guidelines for ADM-assisted implant-based breast reconstruction. Inclusion criteria includes a body mass index (BMI) of <35 kg/m2, no previous radiotherapy, an estimated mastectomy weight of <500 g and a good subcutaneous layer (pinch test>1 cm). The technique of prepectoral implant based breast reconstruction has been described by the author previously.^9^

We looked at the incidence of rippling in our centre. All consecutive patients had prepectoral implant-based breast reconstruction using a pre-shaped Braxon® mesh: a porcine derived acellular dermal matrix (ADM) that is 0.6 mm thick.^9^^,^^10^ All patients in this series had fixed volume silicone implant with an average volume of 360 mL (range: 150-540 mL). Basic demographics of our series are noted in Table 2. Patients had a median follow up of 12 months (range 8-38 months). The patients had a median BMI (Kg/m2) of 26.4 (20.3–34.8).

**Table 2:** Basic demographics of our series

**Patients (n=50)**	**Frequency**
Braxon implants	60
Unilateral	42
Bilateral	9
Median Age (years)	55 (range: 40-71)
BMI (kg/m2)	26.4 (20.3-34.8)
Median size of implant (g)	360 (175-480)
Median follow up (months)	12.4 (4-21)

## RESULTS

We looked at the first 50 consecutive patients who underwent prepectoral implant based breast reconstruction. In our experience, 45 patients (6 bilateral, 90%) had grade 1, 3 patients (2 bilateral, 6%) had grade 2, 1 patient (unilateral, 2%) had grade 3 and 1 patient (1 bilateral, 2%) had grade 4 rippling. The observed rippling was seen more often in patients with low BMI<20 and in those who low subcutaneous fat preoperatively (pinch test<2 cm). All patients with rippling were offered correction of which only 10% underwent treatment. Two patients with grade 2, One patient with grade 3 underwent lipomodelling, while the patient with grade 4 underwent exchange to a larger implant and lipomodelling.

## DISCUSSION

Breast cancer appears to be the most frequent cancer among women with an increase in the number of patients having mastectomy with immediate implant based reconstruction each year. Historically, subcutaneous implant based reconstruction was associated with poor cosmetic outcome including rippling and visible implant contours.^11^^,^^12^ As such, there was a paradigm shift towards submuscular implant based breast reconstruction. However, over the recent years, prepectoral breast reconstruction has regained its popularity due to the problems associated with submuscular implant based reconstruction, that largely being animation deformity.^3^

**Fig. 2:**
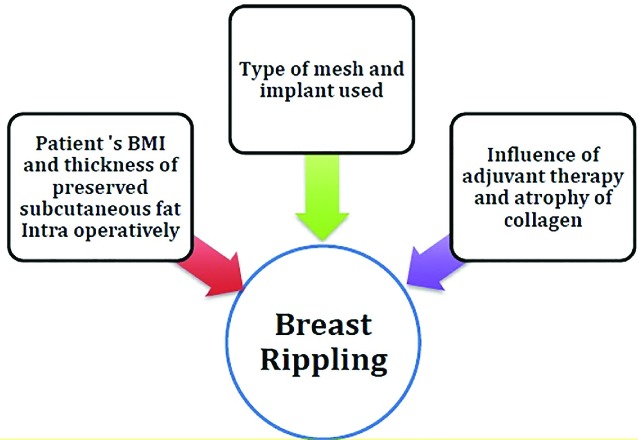
Factors that can influence the occurrence of rippling in prepectoral reconstruction

Prepectoral implant based reconstruction has been shown to provide a good cosmesis with minimal pain, and due to its plane, avoid animation deformity.^12^ However, the same plane gives rise to rippling, an unwanted side effect that can be observed over time. The current literature reports the incidence of rippling to lie between 0-35% (Table 3). In our series, 10% of patients with rippling underwent intervention. The factors that could constitute towards rippling are shown in Figure 2.

**Table 3:** Summary of studies reporting rippling

**Author**	**Year**	**No. of patients/cases **	**Immediate vs delayed reconstruction**	**Type of reconstruction (one or two stage)**	**Implant **	**Coverage technique**	**Follow-up (months)**	**Rippling (%)**
Berna *et al. *(11)	2014	19/25	Immediate	One stage	NS	ADM (Braxon)	7-20	0
Bernini *et al.* (7)	2015	34/39	Immediate	One stage	NS	Mesh (TiLoop)	16-40	9
Kobraei *et al.* (8)	2016	13/23	Immediate	One stage	Silicone gel round and anatomical	Vicryl mesh±ADM	6-18	7
Downs *et al.* (9)	2016	45/79	Immediate	One stage	Silicone gel anatomical	ADM (AlloDerm or FlexHD)	12.7-33.5	35.1

Indeed, subcutaneous cover influences the degree of rippling and is often less prevalent in the patients with a high BMI and the ones with preserved subcutaneous fat during surgery.^11^ The type of implant could have a major influence, greater rippling is observed with saline implants as well as in non-textured implants, when correcting rippling, the above factors need be considered.^13^ Other factors that need to be taken into consideration for planning a prepectoral implant reconstruction includes skin excess and the volume of the implant to skin ratio. It is vital to avoid skin excess and choose the appropriate implant to the skin ratio to eliminate rippling.

To avoid rippling in the patients with low BMI, Salibian *et al.* recommended closing the inframammary incision under moderate tension to create the subcutaneous pocket base smaller than the implant. They also used low-volume (<30 ml) fat injections.^14^ Lipomodelling is often used to correct rippling and can be carried out in stages with fat harvested and stored but injected over a period of time. Along with lipomodelling, a number of adjunct procedures can be employed: if saline implants were initially used then converting to gel based implants may provide some improvement.^15^

The addition of another ADM to the thinned out flap can provide thickness to the upper pole making the implant less visible reduce rippling.^15^^,^^16^ Combination of ADM and lipomodelling (total envelope fat grafting technique) restores the thickness of mastectomy skin flaps resulting in improvement in the aesthetic results.^17^ Finally, a capsulorrhaphy can be performed with exchange of implants along with fat injections. Based on our experience, we have developed a grading system of the implant rippling and its management depending on the grade (Table 2).

Patients with grade 2 and grade 3 rippling were corrected with lipomodelling. The fat is grafted from the patient’s abdomen, thigh, or knee areas. Patients with grade 4 rippling benefit from higher volume lipomodelling with or without exchange of implant. Further follow up of the patients after the fat injections is important with re-evaluation and possible repeat of lipomodelling procedure, if necessary, in 3-6 months. This should be discussed with the patient before the first lipomodelling session to manage the patient’s cosmetic expectations. 

Indeed, further longitudinal evaluation is required to validate our results. In our series we mainly used lipomodelling while other adjuncts such as change of implants, tightening of excess skin can be offered to correct rippling. We also postulate that using an expander inflated with air may cause less tissue atrophy as it is lighter and results in less stretching of the skin.^18^ However, formal evaluation of this is required. Prepectoral or muscle sparing implant-based breast reconstruction is a minimally invasive method of breast reconstruction. This new technique brings a new choice in implant-based breast reconstruction with preservation of normal anatomy. However, rippling is an adverse effect associated with this technique and patients should be well informed.

## CONFLICT OF INTEREST

The authors declare no conflict of interest.
